# Donor and recipient genetic variants in *NLRP3* associate with early acute rejection following kidney transplantation

**DOI:** 10.1038/srep36315

**Published:** 2016-11-07

**Authors:** Mark C. Dessing, Jesper Kers, Jeffrey Damman, Gerjan J. Navis, Sandrine Florquin, Jaklien C. Leemans

**Affiliations:** 1 Department of Pathology, Academic Medical Center, University of Amsterdam, Amsterdam, the Netherlands; 2 Department of Internal Medicine, Division of Nephrology, University Medical Center Groningen, University of Groningen, Groningen, the Netherlands; 3 Department of Pathology, Radboud University Nijmegen Medical Center, Nijmegen, the Netherlands

## Abstract

NLRP3 (NOD-like receptor family, pyrin domain containing 3) is a member of the inflammasome family and is of special interest in renal disease. Experimental studies have shown that Nlrp3 plays a significant role in the induction of renal damage and dysfunction in acute and chronic renal injury. However, the role of NLRP3 in human renal disease is completely unknown. From a retrospective cohort study, we determined in 1271 matching donor and recipient samples if several *NLRP3* single nucelotide polymorphisms (SNPs) were associated with primary non-function (PNF), delayed graft function (DGF), biopsy-proven acute rejection (BPAR) and death-censored graft and patient survival. *NLRP3* gain-of-function SNP (rs35829419) in donors was associated with an increased risk of BPAR while *NLRP3* loss-of-function SNP (rs6672995) in the recipient was associated with a decreased risk of BPAR in the first year following renal transplantation (HR 1.91, 95% CI 1.38–2.64, P < 0.001 and HR 0.73, 95% CI 0.55–0.97, P = 0.03 resp.). *NLRP3* SNPs in both donor and recipient were not associated with PNF, DGF, graft survival or patient survival. We conclude that genetic variants in the *NLRP3* gene affect the risk of acute rejection following kidney transplantation.

Improving long-term renal allograft survival remains a major challenge and allograft longevity is affected by factors including donor type and age, kidney preservation method, ischemia-reperfusion injury (IRI) and occurrence of acute rejection^1^. Because the pool of living (un)related donor kidneys (LD) is limited, the majority of renal allografts are recovered from deceased brain dead and deceased cardiac dead donors. Unfortunately, the latter two groups display more IRI and inferior transplant outcome compared to LD^2^. IRI leads to necrosis, local and systemic immune activation and it contributes to delayed graft function (DGF), which in turn is associated with higher rates of acute rejection and affect graft survival. Innate immunity plays an important role in the mechanisms underlying IRI. Inflammasomes are innate immune sensors and monitor for signs of infection and tissue damage^3^,^4^. Protein-complex formation of inflammasome member NLRP3 (NOD-like receptor family, pyrin domain containing 3) with ASC (Apoptosis-associated Speck-like protein containing a Caspase activation and recruitment domain) and Caspase-1 leads to formation of active IL-1β, IL-18 and IL-33 and, through non-canonical inflammasome signalling, to a programmed form of cell death called pyroptosis^3^,^4^,^5^,^6^,^7^. NLRP3 is the best-characterized inflammasome and is of special interest in renal diseases. NLRP3 is able to induce an inflammatory response upon activation by endogenous stress ligands called damage-associated molecular patterns (DAMPs), which are released following tissue injury, including IRI. In addition, NLRP3 is highly expressed in murine and human renal epithelial cells and leukocytes and increasing amounts of experimental data show that Nlrp3 deficiency prevents renal dysfunction, damage and inflammation in several murine acute and chronic renal disease models^8^,^9^,^10^,^11^,^12^. Genotyping of SNPs (single nucleotide polymorphisms) is frequently used to determine the effects of genetic variations in the context of human diseases. Determination of *NLRP3* SNPs in donor and recipient DNA offers an unique opportunity to characterize the effects of transplant-associated NLRP3 compared to the host’s infiltrating leukocyte-associated NLRP3, respectively. So far it is known that *NLRP3* SNPs with a gain-of-function (GOF) are related to several auto-inflammatory disorders due to an enhanced inflammatory state in these patients^13^,^14^,^15^,^16^. Currently, it is unknown if *NLRP3* SNPs are related to human renal diseases or patient outcome after solid organ transplantation^5^. Therefore, we determined *NLRP3* SNPs in a large cohort of matching donors and recipients and investigated their association with renal allograft and patient outcomes.

## Results

### *NLRP3* SNP distribution in donors and recipients

Baseline characteristics of donors and recipients are displayed in Table 1. In our cohort, genotypic distribution of *NLRP3*-related SNPs in donor and recipient were comparable to previous general/Caucasian population distributions (Supplementary Table S1 and www.ensemble.org). None of the SNPs deviated significantly from a Hardy-Weinberg equilibrium (all P > 0.46 after Holm-Bonferroni correction). In the combined model (A/A vs. A/a + a/a) genotype distribution of *NLRP3* SNPs were comparable between donors and recipient and SNPs were not considered as a potential risk factor for diseases of the native kidneys. SNPs with minor allele frequency less than 1% were left out from further analysis (N = 3).

### *NLRP3* SNPs are not associated with delayed graft function, primary non-function, graft survival or patient survival

Delayed graft function (DGF) occurred in 415/1271 patients (33%) of which 60/415 resulted in primary non-functioning (PNF) of the graft (14% of DGF, 5% of total patients). None of the 3 *NLRP3* SNPs in either donor or recipient were correlated to the occurrence of DGF or PNF (Table 2). After stratification by donor type (living and cadaveric), again none of the *NLRP3* SNPs were significantly associated with DGF or PNF (Supplementary Table S2). Median overall graft survival was 5.5 years (interquartile range 2.9–8.9 years). The overall cumulative incidence of death-censored graft failure was 215/1271 (17%) of which 136/215 due to rejection (63% of graft failure, 11% of total). We did not observe a significant association between any of the *NLRP3* SNPs, in either donor or recipient, with death-censored graft failure (Table 2). The cumulative incidence of patient mortality was 220/1271 (17%). Donor and recipient *NLRP3* SNPs were not associated with patient survival (Table 2).

### Genetic variants in *NLRP3* are a risk factor for biopsy-proven acute rejection

**Figure 1:**
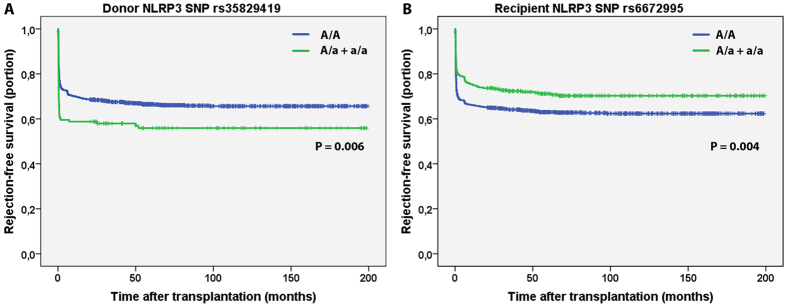
Kaplan-Meier curves for biopsy-proven acute rejection-free survival in donor and recipient NLRP3 single nucleotide polymorphisms. Kaplan-Meier curves for rejection-free survival of (**A**) donor *NLRP3* single nucleotide polymorphism (SNP) rs35829419 and (**B**) recipient *NLRP3* SNP rs6672995. The homozygous dominant genotypes (A/A) are depicted in blue and the combination of the heterozygous (A/a) and homozygous recessive (a/a) genotypes are shown in green. The P-value is calculated by log-rank testing.

The overall cumulative incidence of biopsy-proven acute rejection (BPAR) was 34% (433/1271). The *NLRP3* SNP rs35829419 (GOF) in the donor (A/a + a/a) was significantly associated with an increased risk for BPAR in the complete follow-up (HR 1.48, 95% CI 1.12–1.96, P = 0.006; Table 3 and Fig. 1), which was observed in cadaveric, but not in living donors after stratification for donor type (cadaveric donor HR 1.58, 95% CI 1.16–2.16, P = 0.004 vs. living donor HR 1.16, 95% CI 0.62–2.18, P = 0.64). *NLRP3* SNP rs6672995 (LOF) in the recipient (A/a + a/a) was significantly associated with a decreased risk for BPAR in the complete follow-up (HR 0.72, 95% CI 0.58–0.91, P = 0.005; Table 3 and Fig. 1) which was observed in cadaveric, but not living donor after stratification for donor type (cadaveric donor HR 0.69, 95% CI 0.53–0.90, P = 0.005 vs. living donor HR 0.83, 95% CI 0.53–1.30, P = 0.42). From the Kaplan-Meier curves we could observe that BPAR occurred particularly within the first year after transplantation (Fig. 1). Therefore, we also stratified the patients in those who experienced a BPAR within the first 12 months after transplantation (early BPAR, N = 393 events) and those who experienced their first episode of BPAR later than 12 months after transplantation (late BPAR, N = 40 events). *NLRP3* SNP rs35829419 in the donor was significantly associated with early, but not late BPAR (early BPAR HR 1.54, 95% CI 1.15–2.05, P = 0.003 vs. late BPAR HR 0.82, 95% CI 0.25–2.67, P = 0.74; Table 3). Similarly, *NLRP3* SNP rs6672995 in the recipient was associated with early, but not late BPAR (early BPAR HR 0.68, 95% CI 0.54–0.87, P = 0.002 vs. late BPAR HR 1.16, 95% CI 0.60–2.25, P = 0.66; Table 3). Little differences in donor type distribution were observed for rs6672995 SNP allele combinations in recipients (Table S3).

### Donor rs35829419 and recipient rs6672995 *NLRP3* variants are independently associated with biopsy-proven acute rejection

Next, we created multivariate models adjusting for parameters that are known risk factors for BPAR from the literature, including donor and recipient age, recipient gender, type of donation (all without missing values), cold ischemia time (0.4% missing values), number of HLA mismatches (0.6% missing), previous transplantation (0.1% missing), pre-transplant panel reactive antibodies (3.0% missing), pre-transplant dialysis time (3.3% missing) and the use of induction therapy with interleukin-2-receptor antagonists (no missing values). In separate multivariate regression models, donor *NLRP3* SNP rs35829419 (HR 1.37, 95% CI 1.01–1.87, *P* = 0.04; Table 4) and recipient *NLRP3* SNP rs6672995 (HR 0.71, 95% CI 0.55–0.92, *P* = 0.01; Table 4) were independently associated with BPAR on complete follow-up. When we created a multivariate regression model that included both the donor rs35829419 and recipient rs6672995 *NLRP3* SNPs in one analysis, both variants were contributors to the occurrence of BPAR (HR 1.34, 95% CI 0.99–1.83, *P* = 0.06 and HR 0.71, 95% CI 0.55–0.93, *P* = 0.01, respectively; light-gray area Table 4). When we performed mutliple imputations and recalculated the multivariable model including both SNPs, similar results were obtained (HR 1.38, 95% CI 1.04–1.83, P = 0.02 and HR 0.75, 95% CI 0.60–0.94, P = 0.01, respectively). By 10x internal cross-validation, these results were calculated to be 9.9% over-optimistic. Comparable results were obtained when we only investigated the cumulative incidence of BPAR within the first 12 months after transplantation (donor rs35829419 HR 1.42, 95% CI 1.04–1.94, *P* = 0.03 and recipient rs6672995 HR 0.67, 95% CI 0.51–0.89, *P* = 0.005; light-gray area Table 4). Again, when we performed mutliple imputations and recalculated the multivariable model including both SNPs, similar results were obtained (HR 1.41, 95% CI 1.06–1.89, P = 0.02 and HR 0.71, 95% CI 0.56–0.91, P = 0.006, respectively). By 10x internal cross-validation, these results were calculated to be 10.5% over-optimistic. In conclusion, these results indicate that *NLRP3* SNPs rs35829419 (GOF) in the donor and rs6672995 (LOF) in the recipient both contribute to the risk of BPAR, and more specifically to those episodes of BPAR that occur within the first year after transplantation.

## Discussion

The NLRP3 inflammasone is of special interest in renal diseases because of its expression pattern in murine and human kidneys and its detrimental role in experimental models of acute and chronic renal injury^8^,^9^,^10^,^11^,^12^. Thus far, nothing is known about the role of NLRP3 in human renal diseases or solid organ transplantation. Determination of *NLRP3* SNPs in donor and recipient DNA offers a unique opportunity to characterize the differential effects of transplant- and the host’s leukocyte-associated NLRP3 inflammasome. We quantified several *NLRP3* SNPs in a large cohort consisting of >1200 matched donors and recipients. We found that the *NLRP3* gain-of-function SNP rs35829419 in donors associated with an increased risk of acute rejection, whereas on the contrary that the *NLRP3* loss-of-function SNP rs6672995 in recipients associated with a reduced risk of rejection, particularly those episodes that occurred within the first year after transplantation.

*NLRP3* SNPs became of particular interest in human disease because of their association and causal role in several autoinflammatory disorders. Gain-of-function mutations in the *NLRP3* gene (R260W and Q705K) lead to enhanced basal IL-1β production and are linked to Muckle-Well syndrome, familial cold autoinflammatory syndrome and chronic infantile neurologic cutaneous and articular syndrome^13^,^14^,^15^,^16^. Moreover, *NLRP3* SNP Q705K is associated with Crohn’s disease, celiac disease and late-onset Alzheimer disease^17^,^18^,^19^. In contrast, *OR2B11/NLRP3* SNP rs4353135 and *NLRP3* SNP rs6672995 lead to a reduction in basal NLRP3 expression and rs6672995 homozygous variant a/a affects IL-1β production. Both SNPs are therefore considered to cause a loss of the inflammasome’s function^20^. So far, knowledge about the role of NLRP3 in renal diseases is largely acquired from murine studies, where an important role for both tissue- and myeloid-related Nlrp3 in murine IRI was observed^8^,^9^,^10^. The literature suggests that the pathophysiological role of Nlrp3 in murine renal IRI is in part inflammasome-independent^8^,^9^,^21^,^22^,^23^ and might also be related to TGF-β signaling^24^.

We observed an increased risk of rejection with the donor gain-of-function *NLRP3* variant Q705K (rs35829419) and a reduced risk of rejection in recipients carrying the *NLRP3* A71T variant (rs6672995), especially within the first year following transplantation. It is known that episodes of acute rejection that occur early after transplantation are less detrimental to the graft than those that occur at later time-points. Chronic rejection, especially the antibody-mediated variant, is more challenging to treat. Since we observed an association of *NLRP3* SNPs with early, but not late BPAR, this could explain why we did not identify an association with graft loss.

Little is known about the role of NLRP3 in adaptive immunity. Activation of the NLRP3 inflammasome is crucial in IL-1β and IL-18 maturation that in turn, besides their role in innate immunity, play an important part in priming of adaptive (allo)immunity. Both cytokines are involved in development and differentiation of CD4^+^ T-helper lymphocytes (reviewed in refs 4 and 25). Nlrp3 is known to boost adaptive immunity in an inflammasome-dependent manner in respiratory failure^26^, but Nlrp3 deficiency does not affect alum-mediated adjuvant activity in mice challenged with human serum albumin vaccination^27^. To date, it is unknown if NLRP3 is differentially involved in cellular compared to humoral immunity in humans. How gain- and loss-of-function variants in the *NLRP3* gene affect acute rejection remains speculative and is an area of future study. A limitation of our study is the lack of a standardized assays for the determination of donor-specific antibodies over the years and we therefore could not investigate whether the association between the *NLRP3* variants differed for T cell- or antibody-mediated rejection. We were also unable to directly measure the effect of the *NLRP3* variants on inflammasome-related cytokine production. The current study is the first to describe the association between the *NLRP3* variants and acute rejection and we therefore acknowledge that other cohort studies should validate the generalizability and transportability of our findings.

Interestingly, the association of the *NLRP3* gain-of-function specifically in the *donor* and NLRP3 loss-of-function specifically in the *recipient* with the development of rejection suggests that NLRP3 could be a double-edged sword in renal transplantation. NLPR3 is known to be expressed in human myeloid and lymphoid cells, but also in cells of the renal parenchyma, including tubular epithelial cells^9^,^11^,^28^,^29^. Tubular epithelial cells express NLRP3, IL-1β and IL-18 and a gain-of-function of *NLRP3* could result in elevated levels of these cytokines leading to (local) chronic inflammation and chronic kidney injury^11^,^13^,^14^,^15^,^16^,^30^. Additionally, tubular epithelial NLPR3 could also contribute to kidney fibrosis directly. In experimental murine studies for instance, tubular epithelial Nlrp3 was involved in epithelial-mesenchymal transmission (EMT) via TGF-β signaling, which is believed to precede the development of interstitial fibrosis and tubular atrophy^11^. However, besides parenchymal cells, one should keep in mind that also immune cells like resident dendritic cells of donor origin are being transplanted and subsequently present alloimmune epitopes in draining lymph nodes and a gain-of-function of *NLRP3* in these cells could enhance direct allorecognition, resulting in early rejection. *NLRP3* loss-of-function in recipients on the other hand could alter IL-1β, IL-18 and IL-33 production by (infiltrating) leukocytes upon activation, which was shown to be the case for IL-1β^20^. This could lead to a dampened alloimmune response after transplantation. Further experimental studies are required to determine how *NLRP3* is involved in alloimmune recognition.

Given the important role of Nlrp3 in murine models of IRI, one would expect a role for the human *NLRP3* gene in delayed graft function. However, neither donor nor recipient *NLRP3* SNPs associated with delayed graft function or primary nonfunction. This lack of association could be explained by several aspects. Although murine ischemia-reperfusion injury is also characterized by tubular necrosis, there are some distinct differences with delayed graft function, including differences in circulating leukocyte composition, a slightly different renal anatomy, hemodynamic response and the lack of immunosuppressive drugs in the murine IRI model, making a direct extrapolation of murine data to the human clinical setting not always possible^31^. In addition, delayed graft function is a clinical term describing the need for additional renal replacement therapy, which could have other causes besides IRI-related acute kidney injury rejection, drug toxicity and hyperkalemia^32^ making it a heterogeneous surrogate end-point.

In our study, we grouped A/a and a/a genotype together based on low numbers of the latter group but we do not know if A/a is phenotypically different from a/a and how it would affect our study. In donors, we did identify patients with a homozygous recessive rs35829419 variant. For this variant, Verma *et al.* showed that *in vitro*, the rs35829419 mutant monocytes had a significantly higher level of IL1-beta protein at baseline or after stimulation with the pro-pyroptotic substance alum (adjuvant)^16^. Additionally, Roberts *et al.* observed that in the homozygous recessive allele combination, there was an increased plasma concentration of IL1-beta^33^. It is difficult to extrapolate these results to our the findings in our cohort: (1) there was no heterozygous testing in the *in vitro* experiments by Verma *et al.*, (2) they observed that the excretion of IL1-beta was only partly rescued by a pan-caspase inhibitor, indicating potential non-canonical, pyroptotic signalling in their experiments, (3) both studies measured the full IL1-beta and IL18 protein and not the active forms that are cleaved by the inflammasome complex. The loss of function NLRP3 variant rs6672995 was shown to correlate with the NLRP3 expression on the mRNA level^34^. They did not find an association with plasma concentrations of IL1-beta in patients with this variant. In line with this report, Villani *et al.* found that patients who were homozygous for the rs6672995 variant had a lower production of IL1-beta as compared to heterozygous patients^20^. Again no active IL1-beta (or other inflammasome related cytokines), pyroptosis and in this case no patients with a homozygous dominant variant were analyzed and we can therefore not extrapolate these results to our cohort.

In summary, we showed that various gain- and loss-of-function genetic variants in the *NLRP3* gene differentially associate with biopsy-proven acute rejection, specifically within the first year after renal transplantation. These findings imply a complex role for NLRP3 in early allorecognition and rejection, which makes timing of interventions that aim at promoting or reducing NLRP3 inflammasome activity crucial.

## Material and Methods

### Patient genotyping

**Figure 2:**
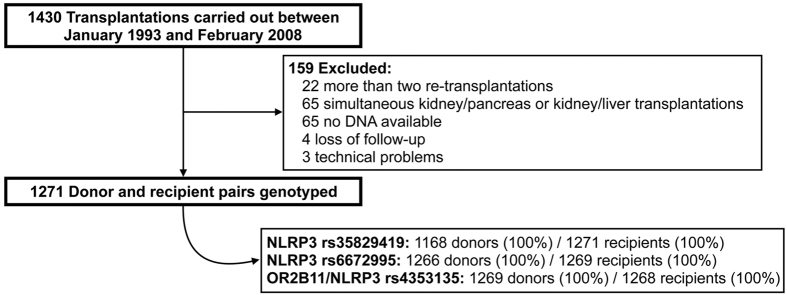
Flowchart of the included patients.

Samples were collected from a retrospective cohort as described before^35^,^36^. Between March 1993 and February 2008, 1271 matching donor and recipient peripheral blood mononuclear cells (PBMCs) were obtained from all available patients who underwent kidney transplantation at the University Medical Center Groningen, The Netherlands. The exclusion criteria were: cases of re-transplantation, combined kidney/pancreas or kidney/liver transplantation, technical problems during surgery, the unavailability of DNA and loss of the patients on follow-up (Fig. 2). The institutional ethical review board of the University Medical Center Groningen approved the study (METc 2014/077). Written informed consent was obtained from all patients. None of the living transplant donors were from a vulnerable population and all living donors provided written informed consent. In case of deceased donation, the donors provided informed consent when they registered their donation status and by law, no additional consent was needed. The study was conducted according to the principles of the declaration of Helsinki. Non-synonymous *NLRP3* SNPs were chosen from NCBI (2010). In addition, we chose the NLRP3/OR2B11 rs4353135 SNP, located downstream of *NLRP3* and upstream of olfactory receptor gene *OR2B11,* which has previously been shown to influence NLRP3 expression levels^20^. Genotyping of the selected SNPs (Supplementary Table 1) was performed using the Illumina VeraCode GoldenGate Assay kit (Illumina, San Diego, CA, USA) according to the manufacturer’s instructions. Genotype clustering and calling were performed using Beadstudio Software (Illumina). The selected SNPs were not in linkage disequilibrium (r^2^ < 0.10; SNP Annotation and Proxy Search, Broad institute).

### DNA isolation and quality control

Peripheral blood mononuclear cells were used to acquire donor and recipient DNA. DNA samples were validated for DNA concentrations using absorbance at 260 nm with a spectrophotometer (ND-1000, NanoDrop) and purity was assessed by 260/280 and 260/230 absorbance ratios. In case of impurity of the sample, repeated isolation attempts were conducted.

### Study end-points

The end-points used in this study were: delayed graft function (DGF, defined as the requirement for dialysis within the first week after transplantation), primary non function (PNF, defined as nonfunctioning of the allograft from transplantation on), time to the first episode of clinical biopsy-proven acute rejection (BPAR), death-censored graft failure (defined as the need for dialysis or re-transplantation) and patient mortality.

### Statistics

Statistical analyses were performed using R version 2.15.2 for Macintosh (www.r-project.org). Two-sided *P*-values below 0.05 were considered statistically significant. As a routine data quality control, the Hardy-Weinberg equilibria for the SNPs were tested. Univariate *P*-values were subjected to Holm-Bonferroni correction for multiple testing. For the association with DGF and PNF, logistic regression models were constructed. For the association with BPAR, (rejection-mediated) death-censored graft failure and patient survival, time-to-event models were created by Cox proportional hazards regression. Data were considered missing (completely) at random and we therefore performed complete-case multivariable regression on 1014/1271 (79.8%) of patients with complete multivariate data available. To validate the effect of missing values on our multivariate models, we reperformed the Cox regression analyses on M = 20 imputed datasets by multiple imputations by chained equations (MICE). The imputation results from predictive mean matching were visually checked by Kernel density plots. Multivariate Cox models were subjected to 10x internal cross-validation and the decline in slope in the validation cohort was considered as an overestimation measure. Kaplan-Meier survival curves were generated to visualize the association between the genotypes of each SNP in donor and recipient and the time to BPAR. Due to the low numbers of patients with homozygous recessive (a/a) variants, we used co-dominant models in which we compared homozygous dominant (A/A) *versus* heterozygous + homozygous recessive (A/a + a/a) individuals.

## Additional Information

**How to cite this article**: Dessing, M. C. *et al.* Donor and recipient genetic variants in *NLRP3* associate with early acute rejection following kidney transplantation. *Sci. Rep.* **6**, 36315; doi: 10.1038/srep36315 (2016).

**Publisher’s note:** Springer Nature remains neutral with regard to jurisdictional claims in published maps and institutional affiliations.

## Supplementary Material

Supplementary Information

## Figures and Tables

**Table 1:** Baseline characteristics of the whole study group.

Variable	Whole study group N = 1271
Donor characteristics	
Age (mean years ± SE)	44.4 ± 14.4
Male N (%)	645 (51%)
Donortype N (%)	
Living donor	282 (22%)
Cadaveric donor (DBD + DCD)	989 (78%)
Donor cause of death N (%)	
CVA	549 (43%)
Trauma	305 (24%)
Other	135 (11%)
Unknown	282 (22%)
Recipient characteristics	
Age (mean years ± SE)	47.9 ± 13.4
Male N (%)	739 (58%)
Primary kidney disease N (%)	
Glomerulonephritis	271 (21%)
Adult polycyctis kidney disease	167 (13%)
Renal vascular disease	124 (10%)
IgA Nephropathy	98 (8%)
Pyelonephritis	148 (12%)
Diabetic	51 (4%)
Chronic	168 (13%)
Other	244 (19%)
Initial immunosuppression N (%)	
Corticosteroids	1201 (95%)
Mycophenolic acid	907 (71%)
Cyclosporin	1085 (85%)
Azithioprin	72 (6%)
Tacrolimus	97 (8%)
Sirolimus	38 (3%)
Induction therapy	
ATG	103 (8%)
Anti-CD3 moab	19 (2%)
Interleukin-2 RA	199 (16%)
Transplant number N (%)	
First	1142 (90%)
Second	128 (10%)
Transplant characteristics	
Cold ischemia time (mean hours ± SE)	
Living donor	2.7 ± 1.9
Cadaveric donor	20.7 ± 6.5
HLA no. of 0 mismatches N (%)	241/1050 (23%)
Cause of graft loss N = 212 (%)	
Rejection	132 (62%)
Technical problems	37 (17%)
Primary recurrent disease	16 (8%)
Primary non-viable	12 (6%)
Infection	3 (1%)
Other	12 (6%)

DBD = deceased brain death, DCD = deceased cardiac death, CVA = cerebrovasculair accident, ATG = antithymocyte globulin, moab = monoclonal antibody, RA = receptor antagonist, SE = standard error.

**Table 2:** Association of *NLRP3* single nucleotide polymorphism with delayed graft function, primary non-function, death-censored graft survival and patient survival.

Gene rs number	Genotype	Donor		Recipient	
		OR (95% CI)	P	OR (95% CI)	P
Delayed graft function (including primary non-function)					
NLRP3 (GOF) rs35829419	A/A (ref)A/a + a/a	1.001.13 (0.77–1.64)	0.53	1.000.72 (0.48–1.04)	0.09
NLRP3 (LOF) rs6672995	A/A (ref)A/a + a/a	1.001.22 (0.95–1.58)	0.12	1.001.07 (0.82–1.38)	0.64
OR2B11/NLRP3 (LOF) rs4353135	A/A (ref)A/a + a/a	1.000.95 (0.75–1.20)	0.64	1.00	0.54
				0.93 (0.73–1.17)	
Primary non-function					
NLRP3 (GOF) rs35829419	A/A (ref)A/a + a/a	1.001.12 (0.46–2.37)	0.78	1.001.15 (0.50–2.34)	0.72
NLRP3 (LOF) rs6672995	A/A (ref)A/a + a/a	1.000.89 (0.48–1.57)	0.71	1.000.98 (0.53–1.72)	0.94
OR2B11/NLRP3 (LOF) rs4353135	A/A (ref)A/a + a/a	1.000.74 (0.44–1.25)	0.26	1.001.08 (0.64–1.81)	0.78
Death-censored graft failure					
NLRP3 (GOF) rs35829419	A/A (ref)A/a + a/a	1.001.12 (0.74–1.70)	0.59	1.000.92 (0.60–1.42)	0.71
NLRP3 (LOF) rs6672995	A/A (ref)A/a + a/a	1.000.97 (0.72–1.31)	0.86	1.000.83 (0.61–1.15)	0.26
OR2B11 NLRP3 (LOF) rs4353135	A/A (ref)A/a + a/a	1.000.99 (0.76–1.30)	0.94	1.001.11 (0.85–1.45)	0.44
Patient survival					
NLRP3 (GOF) rs35829419	A/A (ref)A/a + a/a	1.00.99 (0.64–1.52)	0.95	1.01.09 (0.72–1.64)	0.68
NLRP3 (LOF) rs6672995	A/A (ref)A/a + a/a	1.00.79 (0.59–1.07)	0.13	1.01.24 (0.93–1.66)	0.14
OR2B11 NLRP3 (LOF) rs4353135	A/A (ref)A/a + a/a	1.01.01 (0.78–1.32)	0.93	1.01.15 (0.88–1.50)	0.30

Homozygous dominant (A/A) is considered reference group (ref) and compared to heterozygous + homozygous recessive (A/a + a/a), GOF = gain of function, LOF = loss of function, OR = odds ratio, CI = confidence interval, HR = hazard ration.

**Table 3:** Association of *NLRP3* single nucleotide polymorphism with biopsy proven acute rejection.

Gene rs number	Genotype	Donor		Recipient	
		HR (95% CI)	P	HR (95% CI)	P
Biopsy-proven acute rejection					
NLRP3 (GOF) rs35829419	A/A (ref)A/a + a/a	1.001.48 (1.12–1.96)	0.006	1.000.93 (0.68–1.26)	0.62
NLRP3 (LOF) rs6672995	A/A (ref)A/a and a/a	1.001.11 (0.90–1.36)	0.32	1.00**0.72 (0.58–0.91)**	**0.005**
OR2B11/NLRP3 (LOF) rs4353135	A/A (ref)A/a + a/a	1.001.06 (0.87–1.28)	0.58	1.001.04 (0.96–1.25)	0.70
Biopsy-proven acute rejection, ≤12 months after transplantation (N = 391)					
NLRP3 (GOF) rs35829419	A/A (ref)A/a + a/a	1.001.54 (1.15–2.05)	0.003	1.000.88 (0.64–1.22)	0.45
NLRP3 (LOF) rs6672995	A/A (ref)A/a + a/a	1.001.09 (0.88–1.35)	0.42	1.00**0.68 (0.54–0.87)**	**0.002**
OR2B11/NLRP3 (LOF) rs4353135	A/A (ref)A/a + a/a	1.001.06 (0.87–1.30)	0.56	1.001.06 (0.87–1.29)	0.58
Biopsy-proven acute rejection, >12 months after transplantation (N = 851)					
NLRP3 (GOF) rs35829419	A/A (ref)A/a + a/a	1.000.82 (0.25–2.67)	0.74	1.001.30 (0.54–3.09)	0.56
NLRP3 (LOF) rs6672995	A/A (ref)A/a + a/a	1.001.24 (0.64–2.41)	0.52	1.001.16 (0.60–2.25)	0.66
OR2B11/NLRP3 (LOF) rs4353135	A/A (ref)A/a + a/a	1.000.93 (0.50–1.73)	0.82	1.000.83 (0.44–1.54)	0.55

Homozygous dominant (A/A) is considered reference group (ref) and compared to heterozygous + homozygous recessive (A/a + a/a). GOF = gain of function, LOF = loss of function, HR = hazard risk, CI = confidence interval.

**Table 4:** Multivariate regression for the association of *NLRP3* single nucleotide polymorphism with biopsy proven acute rejection.

Gene rs number	Genotype	Source	HR (95% CI)	P
Biopsy-proven acute rejection				
NLRP3 (GOF) rs35829419	A/A (ref)A/a + a/a	Donor	1.0**1.37 (1.01–1.87)**	**0.04**
NLRP3 (LOF) rs6672995	A/A (ref)A/a + a/a	Recipient	1.0**0.71 (0.55–0.92)**	**0.01**
NLRP3 (GOF) rs35829419	A/A (ref)A/a + a/a	Donor^a^	1.01.34 (0.99–1.83)	0.06
NLRP3 (LOF) rs6672995	A/A (ref)A/a + a/a	Recipient^a^	1.00.71 (0.55–0.93)	**0.01**
Biopsy-proven acute rejection, ≤12 months after transplantation				
NLRP3 (GOF) rs35829419	A/A (ref)A/a + a/a	Donor	1.0**1.91 (1.38–2.64)**	**<0.001**
NLRP3 (LOF) rs6672995	A/A (ref)A/a + a/a	Recipient	1.0**0.73 (0.55–0.97)**	**0.03**
NLRP3 (GOF) rs35829419	A/A (ref)A/a + a/a	Donor^a^	1.0**1.42 (1.04–1.94)**	**0.03**
NLRP3 (LOF) rs6672995	A/A (ref)A/a + a/a	Recipient^a^	1.0**0.67 (0.51–0.89)**	**0.005**

Homozygous dominant (A/A) is considered reference group (ref) and compared to heterozygous + homozygous recessive (A/a + a/a). All models are adjusted for donor age, donation type, recipient age, recipient gender, cold ischemia time, number of HLA A/B/DR mismatches between donor and recipient prior transplantation, pre-transplant panel reactive antibody%, pre-transplant dialysis time and induction therapy with IL2-receptor antagonists.

^a^Light-gray area indicates the multivariable models that include both NLRP3 SNP (donor rs35829419 and recipient rs6672995) together with the aforementioned parameters. GOF = gain of function, LOF = loss of function, HR = hazard risk, CI = confidence interval.
