# Supplementary material for: Donor and recipient genetic variants in NLRP3 associate with early acute rejection following kidney transplantation
Source: Sci Rep. 2016 Nov 7;6:36315. doi: 10.1038/srep36315 (PMC5098240; doi:10.1038/srep36315)
Supplement: Supplementary Information [file srep36315-s1.doc]

**Donor and recipient genetic variants in *NLRP3* associate with early acute rejection following kidney transplantation**

Mark C. Dessing^a,*,1^, Jesper Kers^a,1^, Jeffrey Damman^a^, Gerjan J. Navis^b$^ , Sandrine Florquin^a,c^  and Jaklien C. Leemans^a^

^a^ Department of Pathology, Academic Medical Center, University of Amsterdam, Amsterdam, the Netherlands, ^b^ Department of Internal Medicine, Division of Nephrology, University Medical Center Groningen, University of Groningen, Groningen, the Netherlands, ^c^ Department of Pathology, Radboud University Nijmegen Medical Center, Nijmegen, the Netherlands.

* corresponding author, ^1^ Authors contributed equally to manuscript.

$ On behalf of the REGaTTA (REnal GeneTics TrAnsplantation) Groningen group.

Supplementary Table S1: Genotype detail of *NLRP3* single nucleotide polymorphism in donor and recipient

|  |  | | | | | **Donor** | | | **Recipient** | | |
| --- | --- | --- | --- | --- | --- | --- | --- | --- | --- | --- | --- |
| **Gene** | | **rs number** | **Function**  **(Amino acid alteration)** | **Phenotype** | **FS score** | **Genotype**  **A/A (%)** | **Genotype**  **A/a (%)** | **Genotype**  **a/a (%)** | **Genotype**  **A/A (%)** | **Genotype**  **A/a (%)** | **Genotype**  **a/a (%)** |
| NLRP3 | | rs76291085 | Missense (T195RK) | unknown | Unknown | 99.9 | 0.1 | - | 99.8 | 0.2 | - |
| NLRP3 | | rs35829419 | Missense (Q705K) | GOF ^16^ | 0.407 | 89.4 | 10.6 | - | 88.1 | 11.4 | 0.5 |
| NLRP3 | | rs6672995 | Regulatory region | LOF ^20^ | 0.500 | 71.2 | 26.4 | 2.4 | 72.9 | 25.5 | 1.6 |
| NRLP3 | | rs71642413 | Missense (C276W) | unknown | unknown | 99.9 | 0.1 | - | 99.1 | 0.9 | - |
| NLRP3 | | rs6677787 | Missense (A71T) | unknown | 0.749 | 99.9 | - | 0.1 | 100.0 | - | - |
| OR2B11/NLRP3 | | rs4353135 | upstream variant 2KB | LOF ^20^ | 0.500 | 44.6 | 45.1 | 10.3 | 50.1 | 41.2 | 8.7 |

Homozygous dominant (A/A), heterozygous (A/a), homozygous recessive (a/a). LOF = loss of function. Functional significance score (FS score) incorporates: functional effects of SNPs, predicted at the splicing, transcriptional, translational and post-translational level. According to F-SNP, rs35829419 has an effect on splicing regulation, rs6672995 and rs4353135 are predicted to effect transcriptional regulation and rs6677787 most likely affects protein coding.

Supplementary Table S2: Association of *NLRP3* single nucleotide polymorphism with delayed graft function and primary non-function after stratification for donor type (living vs cadaveric)

| **Living Donor** | | | | | |
| --- | --- | --- | --- | --- | --- |
| **Gene**  **rs number** | **Genotype** | **Donor** | | **Recipient** | |
|  |  | **OR (95% CI)** | **P** | **OR (95% CI)** | **P** |
| **Delayed graft function (including primary non-function)** | | | | | |
| NLRP3 (GOF)  rs35829419 | A/A (ref)  A/a + a/a | 1.0  1.37 (0.29-6.39) | 0.69 | 1.0  1.13 (0.24-5.24) | 0.88 |
| NLRP3 (LOF)  rs6672995 | A/A (ref)  A/a + a/a | 1.0  1.52 (0.50-4.59) | 0.46 | 1.0  1.08 (0.36-3.25) | 0.90 |
| OR2B11/ NLRP3  (LOF) rs4353135 | A/A (ref)  A/a + a/a | 1.0  0.62 (0.22-1.80) | 0.38 | 1.0  0.47 (0.16-1.40) | 0.18 |
| **Primary non-function** | | | | | |
| NLRP3 (GOF)  rs35829419 | A/A (ref)  A/a + a/a | 1.0  2.98 (0.30-29.59) | 0.35 | 1.0  2.48 (0.25-24.49) | 0.44 |
| NLRP3 (LOF)  rs6672995 | A/A (ref)  A/a + a/a | 1.0  0 | 1.00 | 1.0  0.71 (0.07-6.94) | 0.77 |
| OR2B11/ NLRP3  (LOF) rs4353135 | A/A (ref)  A/a + a/a | 1.0  0 | 1.00 | 1.0  0.32 (0.03-3.11) | 0.33 |
| **Cadaveric Donor** | | | | | |
| **Delayed graft function (including primary non-function)** | | | | | |
| NLRP3 (GOF)  rs35829419 | A/A (ref)  A/a + a/a | 1.0  1.12 (0.74-1.68) | 0.60 | 1.0  0.68 (0.45-1.02) | 0.06 |
| NLRP3 (LOF)  rs6672995 | A/A (ref)  A/a + a/a | 1.0  1.15 (0.87-1.52) | 0.46 | 1.0  1.19 (0.89-1.58) | 0.24 |
| OR2B11/ NLRP3  (LOF) rs4353135 | A/A (ref)  A/a + a/a | 1.0  0.90 (0.69-1.16) | 0.40 | 1.0  0.97 (0.75-1.25) | 0.82 |
| **Primary non-function** | | | | | |
| NLRP3 (GOF)  rs35829419 | A/A (ref)  A/a + a/a | 1.0  1.01 (0.42-2.41) | 0.99 | 1.0  1.07 (0.47-2.42) | 0.87 |
| NLRP3 (LOF)  rs6672995 | A/A (ref)  A/a + a/a | 1.0  0.94 (0.52-1.70) | 0.83 | 1.0  1.06 (0.57-1.94) | 0.86 |
| OR2B11/ NLRP3  (LOF) rs4353135 | A/A (ref)  A/a + a/a | 1.0  0.81 (0.47-1.40) | 0.45 | 1.0  1.18 (0.69-2.02) | 0.55 |

Homozygous dominant (A/A) is considered reference group (ref) and compared to heterozygous + homozygous recessive (A/a + a/a), GOF = gain of function, LOF = loss of function, OR = odds ratio, CI = confidence interval

Supplementary Table S3: Baseline characteristics between genotypes in donor rs35829419 and recipient 6672995

|  | **Donor rs35829419** | | | **Recipient rs6672995** | | |
| --- | --- | --- | --- | --- | --- | --- |
| **Variable** | **A/A**  **(N=1134)** | **A/a + a/a**  **(N=134)** | **P** | **A/A**  **(N=925)** | **A/a + a/a**  **(N=344)** | **P** |
| **Donor characteristics** | | | | | | |
| Age (mean years ± SE) | 44.5±14.4 | 43.8±14.8 | 0.62 | 44.6±14.3 | 43.9±14.7 | 0.45 |
| Male N (%) | 569 (50%) | 74 (55%) | 0.27 | 457 (49%) | 187 (54%) | 0.12 |
| Donor type N (%)    Living donor    Cadaveric donor (DBD+DCD) | 253 (22%)  881 (78%) | 29 (22%)  105 (78%) | 0.86 | 191 (21%)  734 (79%) | 89 (26%)  255 (74%) | **0.05** |
| Donor cause of death N (%)  CVA  Trauma  Other  Unknown | 488 (43%)  267 (24%)  126 (11%)  253 (22%) | 60 (45%)  36 (27%)  9 (7%)  29 (22%) | 0.42 | 406 (44%)  228 (25%)  100 (11%)  191 (21) | 143 (42%)  77 (22%)  35 (10%)  89 (26%) | 0.26 |
| **Recipient characteristics** | | | | | | |
| Age (mean years ± SE) | 48.1±13.5 | 46.2±13.1 | 0.15 | 47.6±13.4 | 48.9±13.5 | 0.12 |
| Male N (%) | 669 (59%) | 68 (51%) | 0.07 | 536 (58%) | 203 (59%) | 0.73 |
| Primary kidney disease N (%)  Glomerulonephritis  Adult polycystis kidney disease  Renal vascular disease  IgA Nephropathy  Pyelonephritis  Diabetic  Chronic  Other | 236 (21%)  150 (13%)  107 (9%)  88 (8%)  134 (12%)  44 (4%)  152 (13%)  223 (20%) | 35 (26%)  16 (12%)  17 (13%)  10 (8%)  13 (10%)  7 (5%)  16 (12%)  20 (15%) | 0.58 | 200 (22%)  118 (13%)  84 (9%)  68 (7%)  109 (12%)  37 (4%)  124 (13%)  185 (20%) | 71 (21%)  49 (14%)  40 (12%)  30 (9%)  39 (11%)  14 (4%)  44 (13%)  57 (17%) | 0.72 |
| Initial immunosuppression N (%)  Corticosteroids  Mycophenolic acid  Cyclosporin  Azithioprin  Tacrolimus  ATG  Anti-CD3 moab  Interleukin-2 RA  Sirolimus | 1068 (94%)  807 (71%)  961 (85%)  66 (6%)  92 (8%)  96 (8%)  17 (1%)  180 (16%)  37 (3%) | 131 (98%)  99 (74%)  122 (91%)  6 (4%)  5 (4%)  7 (5%)  2 (1%)  18 (13%)  1 (1%) | 0.36 | 878 (95%)  662 (72%)  783 (85%)  50 (5%)  77 (8%)  78 (8%)  14 (2%)  135 (15%)  29 (3%) | 321 (93%)  243 (71%)  300 (87%)  22 (6%)  20 (6%)  25 (7%)  5 (1%)  62 (18%)  9 (3%) | 0.72 |
| Transplant number N (%)  First  Second | 1016 (90%)  117 (10%) | 123 (92%)  11 (8%) | 0.44 | 828 (90%)  96 (10%) | 312 (91%)  32 (9%) | 0.57 |
| **Transplant characteristics** | | | | | | |
| Cold ischemia time  (mean hours ± SE)  Living donor  Cadaveric donor | 153±34  1235±381 | 211±336  1303±433 | 0.37  0.09 | 160±132  1239±386 | 156±41  1250±389 | 0.77  0.71 |
| HLA no. of 0 mismatches N (%) | 219/934  (23%) | 22/113  (20%) | 0.69 | 173/781  (22%) | 68/269  (25%) | 0.18 |

Homozygous dominant (A/A) compared to heterozygous + homozygous recessive (A/a + a/a). DBD = deceased brain death, DCD = deceased cardiac death, CVA = cerebrovasculair accident, ATG = antithymocyte globulin, moab = monoclonal antibody, RA = receptor antagonist, SE = standard error.
